# Prevalence and Progression of Cognitive Impairment in Atrial Fibrillation Patients after Treatment with Catheter Ablation or Drug Therapy

**DOI:** 10.1155/2019/7216598

**Published:** 2019-12-14

**Authors:** Tina S. Tischer, Daniel Nitschke, Isabelle Krause, Günther Kundt, Alper Öner, Giuseppe D'Ancona, Erdal Şafak, Hüseyin Ince, Jasmin Ortak, Evren Caglayan

**Affiliations:** ^1^Department of Cardiology, University Hospital, Rostock, Germany; ^2^Institute for Biostatistics and Informatics in Medicine and Ageing Research, University Hospital, Rostock, Germany; ^3^Department of Cardiology, Vivantes Klinikum im Friedrichshain und Am Urban, Berlin, Germany

## Abstract

**Purpose:**

In atrial fibrillation (AF) patients, the effect of catheter ablation or drug therapy on cognition is currently not well investigated. Therefore, we prospectively evaluated AF patients who were either treated 'with drug therapy or underwent catheter ablation for the prevalence and progression of cognitive impairment (CI).

**Methods:**

Randomized participants of the CABANA trial (catheter ablation versus antiarrhythmic drug therapy for atrial fibrillation) and the CASTLE-AF (catheter ablation versus standard conventional treatment in patients with left ventricular dysfunction and atrial fibrillation) study were assessed twice within 6 months by Montreal Cognitive Assessment (MoCA) and Mini-Mental State Examination (MMSE) in our institution.

**Results:**

Forty-five patients from both trials were investigated, and twenty-eight patients received catheter ablation, whereas seventeen patients received drug therapy for rhythm or rate control. The mean age of the twenty-one CABANA trial patients (AF group) was 68.8 ± 7.0 years and of the twenty-four CASTLE-AF study patients (AF/HF group) was 66.8 ± 8.1 years, respectively. Mean time from ablation/randomization to the first interview was 16.8 ± 11 months in the AF group and 28.3 ± 18.4 months in the AF/HF group, respectively. All patients investigated were classified as cognitively impaired with mean cutoff scores <24 by MoCA. Overall, we could not detect significant differences in medically treated versus catheter ablation patients within both groups in mean MMSE or MoCA scores between the first and the second interview (*p* > 0.09). Moreover, patients who received catheter ablation did not show statistically significant differences in the prevalence or progression of cognitive impairment compared to patients who were treated medically, neither within the two groups nor between AF and AF/HF patients (*p* > 0.05).

**Conclusions:**

Prevalence of cognitive impairment in AF patients with comorbidities is substantial. However, in this preliminary prospective study, no apparent impact of AF pretreatment on the prevalence and course of cognitive impairment could be observed.

## 1. Introduction

Atrial fibrillation (AF) is the most common heart rhythm abnormality in clinical practice with age-dependent increasing prevalence. The estimated lifetime risk of AF ranges from 22% up to 26% [1]. This rhythm disorder is associated with a wide range of adverse health outcomes, as those afflicted have a reduced quality of life as well as an increased risk for thromboembolic complications and mortality [1, 2]. In addition, AF confers a 2-fold higher risk for congestive heart failure (HF), and it is suspected that AF may also facilitate progression of HF [1, 3]. Although reduction in cardiac performance remains an important and impactful complication of arrhythmia, there seems to be increasing evidence that AF is also independently associated with cognitive decline and dementia even without clinical history of stroke [1, 4–7]. This effect was revealed in AF patients both with and without coexisting heart failure [6, 8, 9]. However, pathogenesis of cognitive dysfunction in AF patients is still in discussion. Some potential mechanisms, such as cerebral hypoperfusion, inflammation, and hypercoagulability, have been proposed to explain the association between AF and cognitive impairment. Subclinical cerebral infarctions and microbleeds in AF patients play a role in the development of cognitive dysfunction and dementia [10]. Furthermore, cerebral hypoperfusion due to beat-to-beat variability of blood flow or reduced cardiac output in AF patients may be an additional reason for cognitive impairment [11–14]. Furthermore, AF itself is suspected to activate inflammatory processes leading to cognitive impairment due to inflammation-mediated cerebral thrombosis or due to direct effects of the inflammatory markers on the brain [15]. On the contrary, AF ablation increases the risk of microembolisms and cognitive dysfunction in the acute period by periprocedural ischemic strokes and a high incidence of silent cerebral ischemia. However, in the long term, it has been reported that AF ablation is associated with a lower risk of Alzheimer dementia [16]. Until today, no AF treatment has yet been linked to a prevalence of cognitive dysfunction and its development, as the few existing studies dealing with this topic revealed inconsistent results [7, 16–18].

We hypothesized that catheter ablation of AF to maintain the sinus rhythm could prevent or at least decelerate cognitive decline in AF patients with and without heart failure. To evaluate this, we examined AF patients who had been prospectively randomized to different AF therapies at our institution in the CABANA trial (catheter ablation versus antiarrhythmic drug therapy for atrial fibrillation) and the CASTLE-AF study (catheter ablation versus standard conventional treatment in patients with left ventricular dysfunction and atrial fibrillation) [19–23].

The CABANA trial was an investigator-initiated, open-label, multicenter, randomized trial involving 126 centers in 10 countries. It was conducted to detect the effect of catheter ablation versus antiarrhythmic drug therapy on mortality, stroke, bleeding, and cardiac arrest among patients with atrial fibrillation [20]. A total number of 2,204 symptomatic patients with AF aged 65 years or older and patients younger than 65 years with one or more risk factors for stroke were enrolled in this trial from November 2009 to April 2016, with follow-up through December 31, 2017 [21, 23].

The main results of this trial have been published recently and showed that catheter ablation, when compared with drug therapy, is not superior to drug therapy with regard to cardiovascular outcomes at 5 years among patients with AF [21, 23, 24].

The CASTLE-AF study is the largest study comparing rhythm control therapy by catheter ablation to rate control therapy in patients with AF and systolic heart failure [19, 22]. 363 patients were randomized 1 : 1 to either catheter ablation or conventional drug therapy for the treatment of AF with respect to the inclusion criteria of failure or intolerance of antiarrhythmic drug therapy or unwillingness to take antiarrhythmic drugs. It was shown that catheter ablation for atrial fibrillation in patients with heart failure was associated with a significantly lower rate of a composite end point of death for any cause or hospitalization for worsening heart failure than was medical therapy alone [22].

## 2. Methods

### 2.1. Study Population

**Figure 1:**
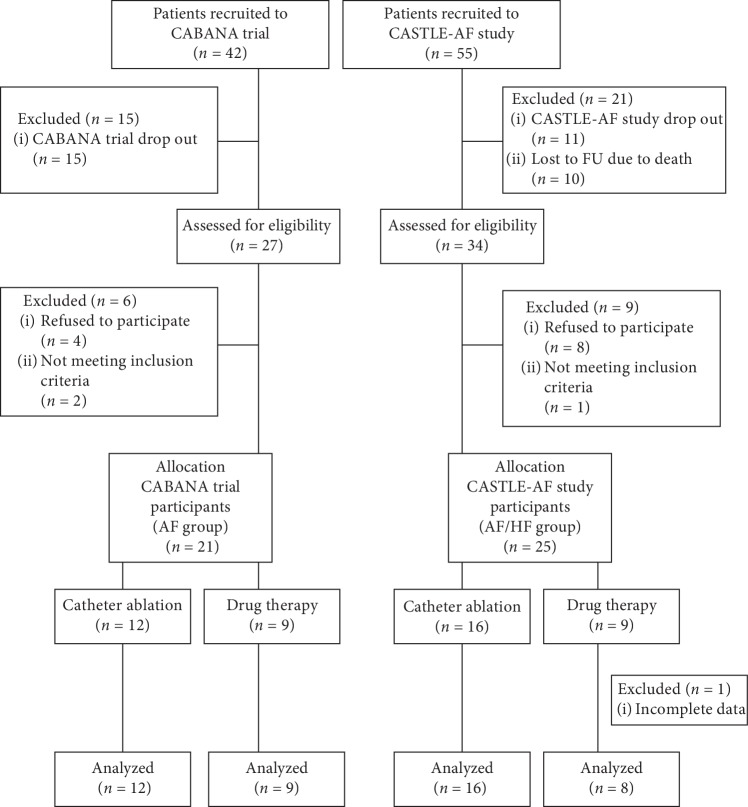
Study flowchart. AF = atrial fibrillation, HF = heart failure, and FU = follow-up.

In 2015 and 2016, we twice requested participants of the CABANA (AF group) and CASTLE-AF (AF/HF group) study to take questionnaires at intervals of 6 months in order to assess cognitive function (Figure 1). For this purpose, all patients completed the Mini-Mental State Examination (MMSE) and Montreal Cognitive Assessment (MoCA) test. Inclusion criteria were age >18 years and written informed consent to participate in the cognitive assessment tests. Exclusion criteria were a medical history of dementia or previous transient ischemic attack or stroke. Patients were enrolled in our study after a period of more than 12 months following randomization to CABANA or CASTLE-AF to avoid periprocedural effects of catheter ablation on cognitive function.

Out of forty-two participants in the CABANA trial (AF group) at our institution, fifteen patients refused to participate. Of the remaining twenty-seven patients, four refused to perform the tests and two were excluded because they met the predefined exclusion criteria. Finally, twenty-one patients from CABANA (AF group) completed our questionnaire (Figure 1).

From the fifty-five CASTLE-AF study patients at our institution, ten participants died and eleven patients dropped out before the beginning of our study. Of the remaining thirty-four patients, eight people refused to complete the questionnaires. We excluded one participant who matched the above exclusion criteria and one participant who died prior to the second questionnaire. Finally, complete data sets with two MMSE and two MoCA tests were eligible for assessment in twenty-four CASTLE-AF patients (AF/HF group) (Figure 1).

All study patients were further analyzed with respect to the absence or prevalence of systolic heart failure and allocated therapy (ablation versus drug therapy). In the AF group, nine patients were treated medically compared to twelve patients who received catheter ablation (Figure 1, Table 1). In the AF/HF group, eight patients received drug therapy, whereas sixteen patients received catheter ablation (Figure 1, Table 1).

This study was approved by the Ethical Commission of the University Medical Centre Rostock (file number A 2014-0075).

### 2.2. Montreal Cognitive Assessment (MoCA) Tool and Mini-Mental State Examination (MMSE)

Cognitive function was assessed by using the MoCA and MMSE test [25, 26]. Both tests take approximately 10 minutes to complete with scores ranging from 0 to 30 points, with lower scores indicating more severe cognitive impairment.

The Mini-Mental State Examination is the most widely used test to evaluate cognitive function with known difficulties in detecting early dementia and mild cognitive impairment, which is considered as a transitional state between dementia and a cognitive impairment beyond that expected for age [25, 27–29]. Amongst others, the MoCA test is considered as an alternative tool to assess cognitive function without presenting the MMSE limitations [26, 28].

Moreover, several differences exist in the reported cutoff points of these scores [25–28]. Recent studies revealed best sensitivity and specificity in detecting even a transitional stage of cognitive impairment between natural aging and dementia with a cutoff point of 24/25 for the MoCA and 27/28 for the MMSE test [28]. In our trial, participants were classified as cognitively impaired by using these cutoff values.

### 2.3. Statistical Analysis

All data were stored and analyzed using the SPSS statistical package 21.0 (SPSS Inc. Chicago, Illinois, USA). Descriptive statistics were computed for continuous and categorical variables. The statistics computed included mean and standard deviation (SD) of continuous variables and are presented as mean ± SD, frequencies, and relative frequencies of categorical factors. Testing for differences of continuous variables between the study groups was accomplished by the 2-sample *t*-test for independent samples or the Mann–Whitney *U* test, as appropriate. Test selection was based on the evaluation of the variables for normal distribution employing the Kolmogorov–Smirnov test. Differences in continuous variables between different time points were investigated using the paired *t*-test or Wilcoxon's rank test for paired data, as appropriate. All *p* values resulted from two-sided statistical tests and values of <0.05 were considered to be statistically significant.

## 3. Results

In summary, we included forty-five patients (twenty-one patients in the AF group and twenty-four patients in the AF/HF group) in our analysis (Figure 1, Table 1). Overall, the majority of the patients in our study population were male (73.3%). In the AF group, the mean age of the patients was 68.8 ± 7.0 years with 9 (42.9%) patients who received medical therapy alone and 12 patients (57.1%) who were treated with catheter ablation in addition. From the 24 patients in the AF/HF group with a mean age of 67.3 ± 7.8 years, 16 patients (66.7%) were in the catheter ablation group, whereas 8 patients (33.3%) were treated with drugs. The first assessment of cognitive impairment with questionnaires in the AF group was made at an average of 16.8 ± 11 months (Table 1). In the AF/HF group, the mean time to first cognitive assessment by MMSE and MoCA was 28.3 ± 18.4 months. The mean intertest intervals were 5.9 ± 2.3 months in the AF patients and 5.7 ± 1.6 months in the AF/HF patients (Table 1).

The AF patients on medical therapy did not show different mean scores for neither MMSE (28.0 vs. 27.8 points; *p*=0.665) nor MoCA (22.4 vs. 23.7 points; *p*=0.342) in the two tests (Table 2). Likewise, we could not detect statistically significant differences in scores for MMSE (26.2 vs. 26.8; *p*=0.232) and MoCA (22.4 vs. 22.9; *p*=0.609) in the subgroup of patients receiving catheter ablation. The results in AF/HF patients were similar with statistically nonsignificant changes in the scores obtained from the questionnaires in both groups of patients investigated. The mean scores between the two time points in medically treated patients were 25.4 vs. 26.3 (*p*=0.087) for MMSE and 21.75 vs. 19.83 (*p*=0.300) for MoCA, respectively. In patients who received catheter ablation therapy, the mean MMSE scores were 26.4 vs. 26.1 (*p*=0.509), and the mean MoCA scores were 22.2 vs. 22.8 (*p*=0.447), respectively (Table 2).

**Figure 2:**
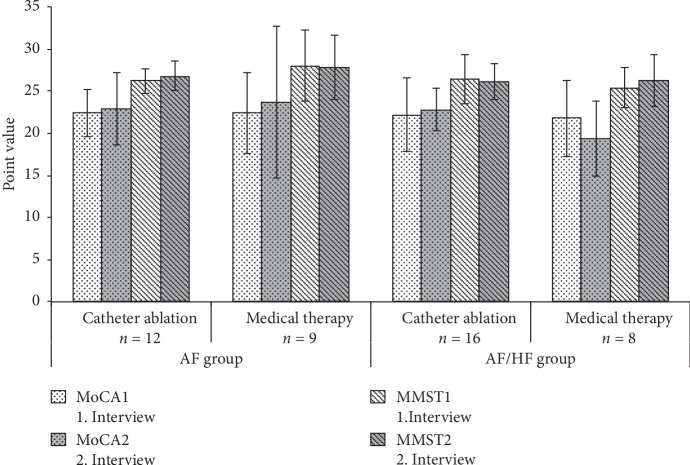
Mean results in MoCA and MMSE test with 6 months intertest interval. AF = atrial fibrillation, HF = heart failure, MoCA = Montreal Cognitive Assessment, and MMSE = Mini-Mental State Examination.

Also, comparing AF and AF/HF patients and their subgroups constituted by AF treatment, no significant differences within the mean MMSE and MoCA tests at the first and the second interview could be found (Table 3, Figure 2). However, all patients were classified as cognitively impaired with mean scores <24 in the MoCA. Moreover, all AF/HF patients as well as all patients treated with catheter ablation in the AF group were classified as cognitively impaired in the presence of mean scores <27 in the MMSE test. Only patients in the AF group who were treated medically reached borderline values in the MMSE test with mean scores of 27.8 and 28 points, respectively. Furthermore, we could not detect statistically relevant differences in the prevalence or progression of cognitive impairment with respect to medical therapy or catheter ablation, neither within the two groups nor between AF and AF/HF patients (*p* > 0.1)(Table 3, Figure 2).

## 4. Discussion

In this study, we evaluated the prevalence and progression of cognitive impairment by using MMSE and MoCA tests in AF patients with and without underlying heart failure with respect to different AF therapies applied. For this purpose, we recruited randomized patients who were participants in the CABANA trial and the CASTLE-AF study. We found that all patients with AF who were recruited for our study had signs of cognitive impairments. However, within an average time interval of 6 months, we could not detect any statistically significant differences in the course of cognitive function, regardless of treatment by medical therapy or catheter ablation.

MMSE and MoCA are brief cognitive screening tests which are usually not used to diagnose dementia or mild cognitive impairment according to the Petersen criteria [29, 30]. However, a positive test may help to detect patients at an early time point who should be further evaluated with neuropsychological testing in order to identify and treat underlying dementia or mild cognitive impairment [29]. The main finding of our study emphasizes that screening for cognitive impairment in elderly AF patients with comorbidities is essential. The prevalence of cognitive impairment measured by MMSE or MoCA in elderly people is high with an occurrence of about 30% up to 46% in those aged >60 years [31, 32]. Yet, the prevalence of cognitive impairment in 100% of our study population was substantial. All AF/HF patients as well as all AF patients treated with catheter ablation in the AF group were considered cognitively impairment with a mean MMSE score <27. Only patients in the AF group who were treated medically reached borderline values with a mean MMSE score from 27.8 to 28 points. Applying the MoCA test, which in general is considered superior to MMSE in detecting mild cognitive impairment in patients above 60 years of age, all study patients fulfilled the criteria of cognitive impairment with mean MoCA scores of less than 24 [28].

The high prevalence of cognitive impairment in our study population is in line with a previous study that analyzed cognitive function in patients with heart failure. Cameron et al. detected mild but potentially significant degrees of cognitive impairment in 73% of patients who were hospitalized with heart failure using MMSE and MoCA tests [33].

A previous study in AF patients estimates the prevalence of mild cognitive impairment or dementia to be about 11% and 28% [34]. Possible reasons for the increased prevalence of cognitive impairment in our cohort compared to the data published by Alonso et al. might be due to differences in the cognitive diagnostic setting used and the evaluation of a larger patient population [34]. A more important reason for the observed difference is that we investigated a sicker population with a high prevalence of relevant comorbidities, which exert an influence on cognitive function of their own. Our patients were not only elderly AF patients but were also multiply diseased (e.g., hypertension, diabetes type 2, heart failure, and arteriosclerotic vascular diseases), as requested for inclusion into CABANA and CASTLE-AF [19, 21]. It is well known that comorbidities such as hypertension, cerebral vasculopathy, diabetes mellitus, or heart failure, are associated with cognitive decline in AF patients [34–36]. Moreover, a strong association between worse cognitive performance and mortality in AF and especially in HF patients was observed [36–39]. Hyunh et al. showed an increase in in-hospital mortality as well as a twofold increase in 30-day death after discharge for heart failure in cognitively impaired patients [39]. Both cognitive impairment as well as AF itself are associated with long-term morbidity and mortality [35, 40].

Based on these observations, we hypothesized that a rhythm control strategy with catheter ablation might be superior for maintaining or even improving cognitive function in AF patients compared to medical therapy alone. The hypothetical positive impact of rhythm control on the course of cognitive function in AF patients is still a matter of debate [35]. A large randomized study failed to present an advantage of rhythm control strategies on cognitive function [17]. Subanalysis of the AFFIRM (Atrial Fibrillation Follow-Up Investigation of Rhythm Management) trial revealed no significant differences in MMSE scores between the rate-control and rhythm-control group in 245 participants over a 3-year follow-up [17]. In contrast, Bunch et al. demonstrated significantly lower rates of incident dementia among patients who were treated with catheter ablation compared to those without ablation in an observational study with a 3-year follow-up [16]. In this study, the authors analyzed medical records of 4,212 patients with AF who underwent catheter ablation and compared them to 16,848 matched patients with medical AF treatment as well as to 16,484 matched control patients [16]. In a retrospective study, Damanti et al. studied 1,082 individuals (aged 65 and older) with AF before hospital admission for any reason. Cognitive performance evaluated as a mean Short Blessed Test (SBT) score was found to be higher in patients with rhythm control than in rate control [41]. Recently, Jin et al. showed in a prospective case control study that catheter ablation of AF at least does not deteriorate cognitive function but rather improves the performance in 1-year follow-up neurocognitive tests, especially in patients suffering from preablation cognitive impairment [18].

In our prospective study, we were not able to detect changes in cognitive function by comparing mean scores in the MMSE and MoCA test in well-characterized AF and AF/HF study patients from CABANA and CASTLE-AF with respect to differences in the therapeutic strategy over a time period of 6 months. A major difference to other studies is the high rate of comorbidities and age in our patient cohort. Our patients were nearly a decade older than those in the study by Jin et al. Also, the number of patients included was relatively small compared to other studies performed.

Our results are in line with the data of the AFFIRM trial which presented no differences in cognitive function by MMSE according to rhythm or rate control therapy over time [17]. Our results suggest that invasive or medical AF therapy might have no influence on progression of cognitive impairment at least between two and three years after ablation or drug therapy for AF. Nonetheless, we hold that the prevalence of cognitive impairment in elderly AF patients with several comorbidities is substantial.

In summary, we present data from a small study population; nevertheless, we included excellently controlled patients from two big prospective randomized trials with an eliminated potential selection bias in comparing invasive AF therapy to medical treatment. On these grounds, our results provide meaningful information.

## 5. Limitations

Our study has some limitations as we failed to assess cognitive function at randomization and the follow-up intertest interval only covered about 6 months. Additionally, mean time between randomization and catheter ablation to first interview partially varied considerably between the groups compared. Further, in AF patients, drug therapy was not uniform, as patients were treated with rhythm and rate control strategies. Apart from that, we have a relatively small sample size since some patients refused to participate in the questionnaires, probably because they were aware of their cognitive impairment and were afraid of performing worse at the tests. Therefore, relevant results were probably missed by our trial.

## 6. Conclusions

Cognitive decline is an independent prognostic factor for worse outcome in AF patients. As our trial presented a substantial prevalence of cognitive impairment in AF patients, we suggest that every elderly AF patient should be assessed for cognitive function by simple screening tools such as the MoCA or MMSE during routine clinical examination. In our study, the course of cognitive function was not significantly different in AF patients with respect to medical therapy or catheter ablation over time. However, further randomized investigations are needed to confirm our results and to understand the pathophysiology of cognitive impairment and the effect of AF treatments on cognition in order to optimize AF therapy.

## Figures and Tables

**Table 1:** Study population.

Population	AF group (aged 68.8 ± 7.0 years) *n* = 21		AF/HF group (aged 67.3 ± 7.8 years) *n* = 24	
	Drug therapy	Catheter ablation	Drug therapy	Catheter ablation
All	9	12	8	16
Females	4	5	0	3
Males	5	7	8	13
Mean time between randomization and catheter ablation to first interview (months)	16.8 ± 11		28.3 ± 18.4	
Mean time between first and second interview (months)	5.9 ± 2.3		5.7 ± 1.6	

AF = atrial fibrillation, HF = heart failure, and SD = standard deviation.

**Table 2:** Results of the study group in twice performed Mini-Mental State Examination and Montreal Cognitive Assessment tool with an intertest interval of 6 months.

Study group	Therapy	*n*	Test	Mean score	Std. deviation	*p*
AF group	Drug therapy	9	First MMSE	28.0	1.50	*p*=0.7
			Second MMSE	27.8	1.72	
			First MoCA	22.4	2.83	*p*=0.3
			Second MoCA	23.7	4.33	
	Catheter ablation	12	First MMSE	26.2	2.92	*p*=0.2
			Second MMSE	26.8	2.08	
			First MoCA	22.4	4.38	*p*=0.6
			Second MoCA	22.9	2.47	

AF/HF group	Drug therapy	8	First MMSE	25.4	4.17	*p*=0.09
			Second MMSE	26.3	3.77	
			First MoCA	21.8	4.89	*p*=0.3
			Second MoCA	19.4	8.99	
	Catheter ablation	16	First MMSE	26.4	2.43	*p*=0.5
			Second MMSE	26.1	3.11	
			First MoCA	22.2	4.51	*p*=0.5
			Second MoCA	22.8	4.49	

AF = atrial fibrillation, HF = heart failure, and SD = standard deviation.

**Table 3:** Results of the AF and AF/HF group according to AF therapy in twice performed Mini-Mental State Examination (MMSE) and Montreal Cognitive Assessment tool (MoCA) with an intertest interval of 6 months.

Test	Study group	Therapy	*n*	Mean score	Std. deviation	*p*
1. MMSE	AF group	Drug therapy	9	28.00	1.5	0.1
	AF/HF group		8	25.4	4.2	
2. MMSE	AF group	Drug therapy	9	27.8	1.7	0.3
	AF/HF group		8	26.3	3.8	
1. MoCA	AF group	Drug therapy	9	22.4	2.8	0.7
	AF/HF group		8	21.8	4.8	
2. MoCA	AF group	Drug therapy	9	23.7	4.3	0.2
	AF/HF group		8	19.4	9.0	
1. MMSE	AF group	Ablation	12	26.2	2.9	0.8
	AF/HF group		16	26.4	2.4	
2. MMSE	AF group	Ablation	12	26.8	2.1	0.5
	AF/HF group		16	26.1	3.1	
1. MoCA	AF group	Ablation	12	22.4	4.4	0.9
	AF/HF group		16	22.2	4.5	
2. MoCA	AF-group	Ablation	12	22.9	2.5	0.9
	AF/HF group		16	22.8	4.5	

AF = atrial fibrillation, HF = heart failure, and SD = standard deviation.

## Data Availability

The patients' data used to support the findings of this study have been deposited in the archives of the Department of Cardiology, University Hospital, Rostock, Germany. Further, the data and results used to support the findings of this study are included.
